# The Book World of Medicine and Science

**Published:** 1898-07-30

**Authors:** 


### The Book World of Medicine and Science.

London and Londoners, 1898. What to See; What to Know; What to Do; Where to Shop; and Practical Hints. Edited by Rosalind Pritchard. (London: The Scientific Press. 1898. Price 2s. 6d.)

Visitors of the better sort, who come to London with the desire of seeing what there is to see and yet do not intend to waste their time, should be very grateful to Miss Rosalind Pritchard for the care she has evidently taken in the preparation of this little book, which will be found an admirable guide-book to all that is best worth doing in the metropolis. The fact is that London is so immense, and the objects of interest that are contained within its bounds are so numerous and so widely scattered, that in the preparation of such a book the selection of the objects to be mentioned is a matter of prime importance. Clearly a little handbook, daintily printed in clear type, provided with wide margins in which the different headings are set out so as to make the way through it clear and easy, cannot contain a complete description of so big a city. Herein, however, lies its virtue. It tells what is wanted and nothing else. Here is the advantage of a guide book written by one who is in the swing, one who is a Londoner *au bout des ongles*, a guide book not written for country parents with long strings of children trying to squeeze as many "free sights" as possible into the week, but for those much more interesting visitors who come up to view that still more interesting and perennially moving sight—how London lives. Under the heading "What to See" come the churches, the statues, the bridges, the exhibitions, and that most important factor in modern London life, the week-end excursions. Under "What to Know," we find the Court, the palaces (with little sketches of their histories), the libraries, societies, charities, books, and, lastly, the restaurants. In telling us "What to Do," Miss Pritchard is not content to give a string of theatres and concert halls, but tells us about the boat race, the four-in-hand meets, the fourth of June at Eton, the cricket matches and regattas, and other things that now enter so largely into every-day conversation wherever people meet. Then there is sport and hotels, and travelling and Sunday dining, and all the rest of it, and there are mysterious chapters which will, no doubt, be of great importance to lady readers, such as "Suitable Dresses for Particular Occasions," "London Ways in 'Smart' Society," "How to Invite Guests—How to Receive Them." Take it altogether, the book is not only interesting, but it is even amusing—a thing that one hardly expects in a guide book—and it occasionally leaves the reader much to ponder on, especially, perhaps, the chapter about "tips." Life in London has, indeed, many aspects, and we can well believe that under the patronage of Miss Rosalind Pritchard the life even of a waiter ought to be a happy one. We should add that the book is very nicely bound in leather, and is by no means suggestive of Baedeker.

Outlines of Practical Surgery. By Walter G. Spencer, M.B., M.S., F.R.C.S., Surgeon to the Westminster Hospital. (London: Baillière, Tindall, and Cox. 1898. Price 12s. 6d. net.)

As the title indicates, this book is confined to practical subjects, it being best, in the opinion of the author, that details of pathology and bacteriology, and such matters as ophthalmic surgery, should be dealt with in special works. Looked at in this light, we have no hesitation in according to the author the highest praise. He has produced a work which from beginning to the end is redolent of practical teaching, and as he possesses the happy knack of making his meaning clear in a few words, the book is one which will be found of great value by all who wish to study practical surgery by itself. If we chose to be hypercritical in regard to so good a treatise, we might suggest that practical surgery ought to be, and indeed is, the logical outcome of a wide knowledge of the whole subject, and that, unless a book like this contains a good deal which cannot be dug out of more complete treatises, it fails to prove the necessity for its existence. Still, we have no doubt that there is a demand for a book of the sort, for many people object to digging, and if there is such a demand it certainly could not have been supplied in a better manner. The book begins with a good description of antiseptics, in which there is an evident leaning to carbolic acid rather than mercuric chloride. It is to be noted that Mr. Spencer does not advocate the very prolonged preparation of the skin of the patient which is advocated by some surgeons; except that the patient has a general bath, the disinfection of the skin does not seem to be commenced until two hours before the operation. In regard to water for washing wounds, he says "clean rain-water or sea-water" may be employed, and he also gives a good hint that "a pressure filter may be attached to a hot-water tap, and if the porous cylinder through which the water is driven be boiled every day or so in soda solution a satisfactory water is the result." Throughout the book, as is only natural, great attention is paid to operative surgery, of the modern position of which it may be taken as giving a fair representation. It should, however, be stated that it by no means deals exclusively with operative nor even manipulative work. It is really a book on the diagnosis and treatment of surgical diseases. A word should be said about the illustrations, which are all of the "black-board" type done with white lines as if by a piece of chalk. They are not particularly elegant, but they serve well to concentrate the attention on the point intended.

Surgical Technics in Hospital Practice. By K. W. Monserrat, M.B., F.R.C.S.E., Assistant Surgeon to the Liverpool Cancer Hospital. (Bristol: John Wright and Co. 1898. Pp. 130. Price 3s.)

This little book deals, in a simple and lucid manner, with those details of surgery and surgical technique which are not to be learnt from books on operative surgery. Antisepsis, asepsis, preparations for operations, after care of patients, all find a place in Mr. Monserrat's pages, and are discussed with that familiarity which is gained only through intimate acquaintance with the best methods. There is little, if anything, to criticise in Mr. Monserrat's directions; we differ from him rarely, and then not in essentials, but in matters in which much latitude of personal opinion may be allowed. Mr. Monserrat does not forget, as so many do, that a hasty swill with carbolic lotion is less cleansing than a housewifely scrub with soap and water; and in dealing with the body he expresses a wise preference for the less irritating antiseptics. The author's choice of dressings is one to which no exception can be taken; the preparations that he advises are admirable, and show great attention to detail. The "Rules for Nurses" form an excellent code for maintenance of discipline and efficiency in the operating theatre. We have noticed no omissions of importance, and only one printer's error—the omission on page 95 of the quantity of water required in making picric acid lotion. Although no doubt surgical technique is best learnt, as a house surgeon, at the feet of a master, this book should not fail to be of great help to many young practitioners, and to older surgeons who are unfamiliar with the newest procedures. For surgical nurses we know of no better book.

